# Bioequivalence of HX575 (recombinant human epoetin alfa) and a comparator epoetin alfa after multiple intravenous administrations: an open-label randomised controlled trial

**DOI:** 10.1186/1472-6904-9-10

**Published:** 2009-05-22

**Authors:** Fritz Sörgel, Ursula Thyroff-Friesinger, Andrea Vetter, Bernhard Vens-Cappell, Martina Kinzig

**Affiliations:** 1 IBMP-Institute for Biomedical and Pharmaceutical Research, Nürnberg, Germany; 2 Clinical Research, Hexal AG, Holzkirchen, Germany; 3 Scope International, Hamburg, Germany

## Abstract

**Background:**

HX575 is a human recombinant epoetin alfa that was approved for use in Europe in 2007 under the European Medicines Agency biosimilar approval pathway. Therefore, in order to demonstrate the bioequivalence of HX575 to an existing epoetin alfa, the pharmacokinetic and pharmacodynamic response to steady state circulating concentrations of HX575 and a comparator epoetin alfa were compared following multiple intravenous administrations.

**Methods:**

An open, randomised, parallel group study was conducted in 80 healthy adult males. Subjects were randomised to multiple intravenous doses of 100 IU/kg body weight of HX575 or of the comparator epoetin alfa three-times-weekly for four weeks. Serum epoetin concentrations were measured using an enzyme-linked immunosorbent assay and pharmacokinetic parameters for the two treatments were compared. The time course and area under the effect curve ratio of haematological characteristics were used as surrogate parameters for efficacy evaluation.

**Results:**

The haematological profiles of both treatments were similar, as determined from their population mean curves and the AUEC_Hb _ratio and 90% confidence interval (99.9% [98.5–101.2%]), the primary pharmacodynamic endpoint of this study. The pharmacokinetic parameters after the treatments showed minor differences after single dosing, but not at steady state doses. After multiple doses, HX575 was bioequivalent to the comparator with respect to the rate and extent of exposure of exogenous epoetin (AUC_τ _ratio and 90% confidence interval: 89.2% [82.5–96.2%]). Study medication was well tolerated with no clinically relevant differences between safety profiles of the treatments. Anti-epoetin antibodies were not detected.

**Conclusion:**

HX575 and the comparator epoetin alfa were bioequivalent at steady state circulating drug concentrations with respect to their pharmacokinetic profile and pharmacodynamic action. This supports the conclusion that HX575 and the comparator epoetin alfa, when administered intraveneously, will be equally efficacious and may be interchangeable as therapy.

## Background

Epoetin is a glycoprotein that stimulates red blood cell (RBC) production [1]. Patients with chronic renal failure have impaired epoetin production, which is the primary cause of their anaemia [2,3]. Human recombinant epoetin or erythropoiesis stimulating agents (ESA) has been shown to stimulate erythropoiesis in anaemic patients with chronic renal failure, both in those who do, and those who do not, require regular dialysis [3-12]. ESA are indicated for treatment of chemotherapy-induced anaemia in cancer patients, and to reduce the need for allogenic blood transfusions in patients with moderate anaemia scheduled to undergo elective surgery [13-15]. In addition, human recombinant epoetin is indicated for patients at high risk for perioperative transfusions with significant, anticipated blood loss.

The aim of this study was to estimate the relative bioavailability and pharmacodynamics of HX575 versus the comparator epoetin alfa at steady state drug concentrations following multiple intravenous administration. The pharmacokinetic profile of epoetin and the haematological effects on haemoglobin were evaluated as a surrogate for the therapeutic efficacy. The primary objective was to assess bioequivalence for the area under the effect curve (AUEC) of haemoglobin. Furthermore, the safety profiles of the two treatments were compared. This article reports the pharmacokinetic, pharmacodynamic and safety data for HX575 and a comparator epoetin alfa.

## Methods

This open, randomised, parallel group study enrolled 80 healthy male volunteers. Eligible subjects were 18–45 years of age, weighing 50–100 kg, physically and mentally healthy as confirmed by an interview, medical history, and clinical and laboratory examination. Other inclusion criteria were: a body mass index of 19–28 kg/m^2^; haemoglobin (Hb) concentrations of 13–15 g/dL; the percentage reticulocytes (Ret, percentage of red blood cells in the reticulocyte stage) ≤ 3% at screening; and normal, or only minor deviation in, iron parameters (iron deficiency was defined as ferritin < 10 ng/mL or Fe/TIBC ratio (transferrin saturation) < 12%). Subjects had to be non-smokers or moderate smokers (≤ 10 cigarettes/day) and abstain from alcohol for 48 h prior to each dose administration. Only male subjects were enrolled in order to minimise inter-subject variability.

Subjects were not eligible if their medical history showed evidence of pre-existing clinically significant cardiac disease or any of the following: clinically significant abnormalities that might influence the metabolism or excretion of the active agent under investigation; increased values (above upper limit of normal range) of reticulocytes, erythrocytes, platelets or serum potassium; baseline serum epoetin level < 30 mIU/mL; use of systemic androgens within two months prior to study start; use of any medication (including over-the-counter treatments) that was not expressively permitted within two weeks prior to study start; epoetin therapy within eight weeks before study start.

An iron supplement (100 mg twice-daily) was administered to all subjects during the study. Except for paracetamol, all other concomitant use of drugs was restricted.

The study was conducted in accordance with the Declaration of Helsinki, Good Clinical Practice and Good Laboratory Practice. The study was approved by an independent ethics committee and all volunteers gave their written informed consent.

Eligible subjects were randomised to receive an intravenous injection of one of two different treatments three-times-weekly for 4 weeks. HX575 treatment consisted of injections of 100 IU/kg body weight of human recombinant epoetin alfa (HX575, Binocrit^®^, Sandoz Pharmaceuticals GmbH, Germany). The comparator treatment consisted of injections of 100 IU/kg body weight of epoetin alfa (Erypo^®^/Eprex^®^, Ortho Biotech, Germany). Both groups received an intravenous injection delivered over a 1 minute period on days 1, 3, 5, 8, 10, 12, 15, 17, 19, 22, 24 and 26. Both HX575 and the comparator epoetin alfa were supplied as 10,000 IU/mL formulations. Subjects fasted for at least 8 h prior to and 2 h after administration.

### Blood sampling

For Ret count, RBC count, Hb concentrations and haemoatocrit (HCT), blood samples of 2.6 mL were drawn: on day 1 at 30, 20, 10 min pre-dose and just before dosing, on days 3, 5, 8, 10, 12, 15, 17, 19, 22, 24 and 26 just before dosing, and on day 29 at 72 h after the last dose. For determination of transferrin, transferrin receptor, ferritin and serum iron concentrations, venous blood samples of 12 mL were drawn on days 1, 12, 19 and 26 at the time of dosing. For anti-epoetin antibodies assays in serum, a blood sample of 3 mL was drawn on days 1, 15 and 29 at the time of dosing. For serum epoetin assays, blood samples of 4 mL were drawn: at 30, 20, and 10 min predose, just before dosing, and at 5, 10, 15, 20, 30, 45 min and 1, 1.5, 2, 3, 4, 5, 6, 8, 10 and 12 h after the first dosing on day 1; on days 8, 15, 19 and 22 at the time of dosing; and at 30, 20, 10 and 0 min before dosing, and at 5, 10, 15, 20, 30, 45 min and 1, 1.5, 2, 3, 4, 5, 6, 8, 10, 12, 16, 24 and 36 h after dosing on day 24.

All sample analyses were performed in the same laboratories. Ret count, RBC count, Hb concentrations and HCT were analysed at LPT Laboratory for Pharmacology and Toxicology KG, Hamburg, Germany. Transferrin, ferritin and serum iron were determined at MDS Pharma Services Central Lab GmbH, Hamburg, Germany. Transferrin receptor was determined at Dres. Fenner & Partner, Hamburg, Germany. Serum anti-epoetin antibodies were measured at Hexal Biotech Forschungs GmbH, Oberhaching, Germany. Serum epoetin concentration measurements were performed at GTF, Gesellschaft für Therapeutische Forschung mbH, Nürnberg-Heroldsberg, Germany.

### Pharmacokinetic assay and evaluation

The concentrations of epoetin in human serum were determined using an enzyme immunoassay kit (EPO-ELISA, Medac GmbH, Germany). The procedure was validated according to international guidelines. During sample analysis, the standard curve was linear between 2.5 mIU/mL and 160.0 mIU/mL for all sequences and the lower limit of quantification for epoetin was 2.5 mIU/mL. Samples expected to exceed the upper limit of the linear range were diluted before analysis with diluent of the enzyme immunoassay kit. The inter-day precision of the control standard of epoetin in human serum was 11.4%.

Pharmacokinetic variables were calculated by non-compartmental analysis using actual data of endogenous epoetin. The primary pharmacokinetic parameter was the epoetin AUC_τ _calculated over a period of 0–36 h. Secondary parameters were AUC_0–12_, C_max_, t_max_, t_1/2_, C_max, ss_, and C_trough_. The treatments were considered to be bioequivalent when the ratio (HX575/comparator) and the 90% confidence interval (CI) for AUC_τ_, C_max, ss_, C_trough_, and t_1/2 _(multiple dose analysis) fell within the acceptance range of 80–125%.

### Pharmacodynamic analyses

The time courses of Hb, RBC, HCT and Ret count were used as surrogate parameters for efficacy. The pharmacodynamic action of epoetin was determined as the area under the total effect curves during 12 dosage intervals in 4 weeks for Hb, RBC, HCT and Ret count, which were calculated by linear trapezoidal integration. The AUEC_Hb _was considered the primary variable. The formulations were considered to be bioequivalent if the 90% CI of the AUEC_Hb _ratio fell within a range of 96.8–103.2%. The rationale for this acceptance range was the following: based on results from a pilot study, the Hb concentration was estimated to change by about 3 g/dL within 4 weeks of the multiple dose regimen of the present study. A difference between the treatments of ± 1 g/dL is considered acceptable, since under clinical conditions tight monitoring of the Hb concentrations is mandatory and no dose adjustment for epoetin is required if the Hb concentrations are stable within a range of ± 1 g/dL. Furthermore, in clinical studies a threshold of -1.0 g/dL Hb has been used as the greatest clinically acceptable difference to demonstrate non-inferiority [16]. The baseline Hb concentration in healthy volunteers was expected to be approximately 14 g/dL. Thus, the expected concentrations at the end of 4 weeks of treatment were approximately 17 ± 1 g/dL. This corresponds to an AUEC_Hb _of (14+17 ± 1)/2 = 15.5 ± 0.5 month*g/dL. The absolute deviation of ± 0.5 from the mean 15.5 translated into a relative acceptable difference for the ratio of (0.5/15.5)*100% = 3.2%, which led to the corresponding acceptance boundaries for the ratio of 96.8–103.2%. For the AUEC ratios (HX575/comparator) and 90% CI of RBC, HCT, and Ret count, no acceptance ranges were derived. Nevertheless, the AUEC ratios and 90% CI were compared in an exploratory fashion with the standard bioequivalence range of 80–125%. Additionally, the time courses of transferrin receptor, transferrin, ferritin and iron concentrations were investigated.

### Detection of anti-rhEPO antibodies

Sera were screened for the presence/absence of anti-rhEPO antibodies using a radioimmuno-precipitation (RIP) assay that was comprehensively validated according to the requirements of the FDA guideline for bioanalytical methods validation [17] and according to ICH guidelines [18,19].

### Safety

Adverse events were obtained from spontaneous reporting by the subjects or from responses to non-leading questions from the clinical staff.

### Statistical methods

Sample size was determined as follows. An inter-individual coefficient of variation of approximately 30% was expected for the (log-transformed) AUEC_Hb _[20]. A minimum of 36 subjects per treatment group had to complete the study in order to determine the relative pharmacodynamic efficiency in terms of the AUEC ratio (HX575/comparator) with adequate precision in an analogous way to a bioequivalence study; the 90% CI of the AUEC_Hb _ratio should fall within a range of 96.8–103.2% with a power of > 80%, provided the true ratio was 100%. For the pharmacokinetic variable AUC_τ_, a coefficient of variation of 20–25% was expected [21]. The precision of the estimate of the AUC_τ _ratio thus was expected to be higher than for the AUEC_Hb _and the sample size was not expected to be critical.

The mean, standard deviation, coefficient of variation, range and median were calculated for each parameter. The geometric mean (GeoM) and the coefficient of variation of the geometric mean (GeoCV) were also determined for concentration related parameters. For AUEC and C_trough _the parametric point estimators for the ratio and the shortest 90% CIs were calculated using the LSMEANS and the root of residual mean squares from the ANOVA of log-transformed data with subsequent exponential transformation [22]. For parameters for which the assumption of normally distributed data was rejected, nonparametric point estimators for the ratios of expected medians of the treatments and the corresponding nonparametric 90% CIs were calculated based on the Mann-Whitney-Wilcoxon statistics using log-transformed data [23,24]. For t_max_, the nonparametric point estimator and the nonparametric 90% CIs for the difference of expected medians were calculated according to the Mann-Whitney-Wilcoxon statistics using the untransformed data.

## Results

Eighty healthy adult males were enrolled: 40 subjects per treatment. Seventy-six subjects completed the study and were available for pharmacodynamic and pharmacokinetic evaluations. The HX575 treatment group was comprised of 37 subjects and the comparator treatment group was comprised of 39 subjects. For the single dose analysis, 29 subjects in the comparator treatment group were analysed, because of the accidental administration of a lower dose in ten subjects at the first application.

Four subjects withdrew from the study: three receiving HX575 treatment and one receiving the comparator treatment. Three subjects withdrew due to adverse events (two subjects receiving HX575 and one receiving the comparator treatment) and for one subject (HX575 treatment) the study was stopped on day 2 due to significant deviations from the normal range of his haematological baseline values on day 1. Demographic data of enrolled subjects are presented in Table 1.

**Table 1:** Demographic characteristics.

**Demographic characteristic**		**HX575** (*n *= 40)	**Comparator** (*n *= 40)
**Age **(year)	Mean	32.6	33.4
	SD	7.4	6.9
	Range	20–46	20–44

**Weight **(kg)	Mean	78.5	78.9
	SD	10.1	9.1
	Range	60–98	59–100

**Height **(cm)	Mean	179.9	180.9
	SD	6.1	5.7
	Range	170–190	166–195

**BMI **(kg/m^2^)	Mean	24.19	24.07
	SD	2.34	2.12
	Range	20.3–28.3	20.7–28.4

*n *= number of subjects receiving treatment, SD = standard deviation.

### Pharmacokinetics

The mean (± SD) pre-dose endogenous epoetin concentration was 8.5 ± 2.4 mIU/mL in the HX575 group and 7.6 ± 1.7 mIU/mL in the comparator group. There were small differences between the two treatments in the concentration-versus-time profiles after a single epoetin dose. Mean epoetin concentrations reached their maxima of 1953 ± 621.2 mIU/mL (HX575) and 2121 ± 505.9 mIU/mL (comparator) within 5 min after the first administration. The mean epoetin plasma concentration had increased by 1944 ± 21.4 IU/mL (HX575) and 2113 ± 505.8 mIU/mL (comparator) from baseline. Twelve hours after the first application, the mean concentrations had dropped to 214.8 ± 77.8 mIU/mL (HX575) and 242.7 ± 71.5 mIU/mL (comparator). The mean trough concentrations after multiple doses of epoetin (Table 2) were similar to the pre-dose baseline concentrations. There was no accumulation and the baseline concentrations appeared almost negligible in relation to the peak concentrations. The AUC_0–12 _after HX575 was approximately 18% lower than after the comparator (GeoM (GeoCV) 8098 mIU/mL*h (44.5%) after HX575 and 9903 mIU/mL*h (33.3%) after the comparator).

**Table 2:** Mean pharmacokinetic parameters after multiple intravenous epoetin doses.

**Treatment**		**AUC_τ _**mIU/mL*h)	**C_max, ss _**(mIU/mL)	**t_max _**(h)	**C_trough _**(mIU/mL)	**t_1/2 _**(h)
**HX575**	Mean	8422	2189	0.086	9.811	4.14
	SD	2419	393.7	0.019	2.728	1.71
	Min	4327	1203	0.083	5.565	1.90
	Median	7930	2180	0.083	9.174	3.48
	Max	19321	3197	0.200	18.61	8.43
	GeoM	8153	2153	-	9.486	3.85
	GeoCV	25.4%	19.3%	-	26.2%	39.0%

**Comparator**	Mean	9224	2262	0.083	9.446	4.74
	SD	1850	422.0	0.000	2.438	2.00
	Min	4977	1272	0.083	5.020	1.31
	Median	9181	2210	0.083	9.161	4.45
	Max	13268	3161	0.083	15.77	11.03
	GeoM	9036	2222	-	9.141	4.37
	GeoCV	21.1%	19.7%	-	26.6%	43.5%

AUC_τ _= area under total concentration curve from 0–36 h at steady state; C_max, ss _= peak serum concentration at steady state; t_max _= time to C_max, ss_; C_trough _= mean pre-dose trough concentration; t_1/2 _= terminal elimination half-life; SD = standard deviation; GeoM = geometric mean; GeoCV = coefficient of variation of GeoM. Number of subjects analysed: *n *= 37 (HX575) and *n *= 39 (comparator).

**Figure 1:**
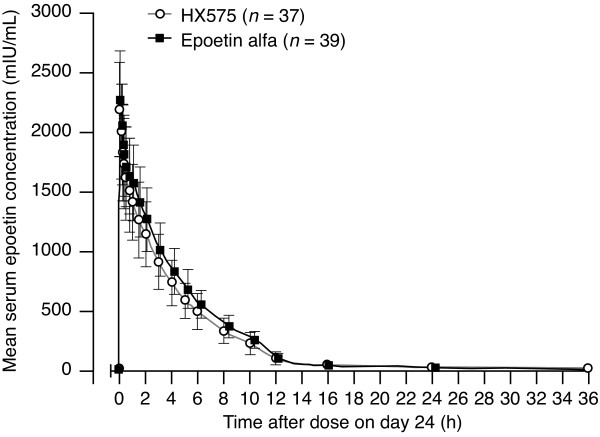
**Mean serum epoetin concentration-versus-time profiles (± SD) after multiple doses not corrected for baseline epoetin levels**. 0 h = time of 11^th ^application on study day 24.

The concentration-versus-time profiles after multiple applications of epoetin were similar for the two treatments (Figure 1). Five minutes after injection, the mean epoetin concentrations reached their peaks (Table 2), which differed by only 3% between both treatments. Mean epoetin levels had increased with 2181 ± 393.5 mIU/mL (HX575) and 2254 ± 421.9 mIU/mL (comparator). The epoetin concentrations had decreased almost to the initial baseline at 36 h after the eleventh application (13.8 ± 23.1 mIU/mL (HX575) and 11.1 ± 4.0 mIU/mL (comparator)). The AUC_τ_, calculated over a time period of 0–36 h, was approximately 10% lower after HX575 than after the comparator (Table 2). The estimates of the terminal elimination half-life (t_1/2_) after single dose (GeoM (GeoCV) 3.97 h (18.6%) after HX575 and 4.03 h (25.5%) after the comparator) and multiple doses (GeoM (GeoCV) 3.85 h (39.0%) after HX575 and 4.37 h (43.5%) after the comparator) were similar, suggesting that the clearance of epoetin was unchanged.

Furthermore, the magnitudes of the C_max, ss _and AUC_τ _after multiple doses were similar to the C_max _and AUC_0–12 _after single dose. The accumulation ratio for C_max _(GeoM of C_max, ss_/C_max_) was 1.2 (HX575) and 1.1 (comparator). The ratio of AUC_τ_/AUC_0–12 _was 0.98 (HX575) and 0.89 (comparator), suggesting that there was no accumulation with respect to the extent of exposure.

To compare the relative bioavailability of the epoetin from HX575 and the comparator, the ratios of the pharmacokinetic parameters were calculated (Table 3). The treatment groups were comparable with respect to the endogenous erythropoietin baseline concentration as indicated by the ratio and 90% CI of 110% [99.5–120.2%]. With the exception of the t_1/2 _ratio, the 90% CIs of the multiple dose ratios fell within the acceptance range for bioequivalence of 80–125% (Table 3). HX575 was therefore pharmacokinetically equivalent to the comparator epoetin alfa following multiple intravenous administrations.

**Table 3:** Ratio (HX575/comparator), 90% confidence intervals, and ANOVA coefficient of variation of pharmacokinetic parameters after multiple epoetin doses.

**PK Parameter**	**Method**	**Ratio **(%)	**90% CI **(%)	**ANOVA-CV **(%)
**AUC_τ_**	MWW-log*	89.2	82.5–96.2	23.3
**C_max, ss_**	MWW-log*	97.5	91.1–104.5	19.5
**C_trough_**	ANOVA-log	103.8	94.0–114.6	26.4
**t_1/2_**	MWW-log*	87.8	75.3–100.0	41.4

AUC_τ _= area under total concentration curve from 0–36 h at steady state; C_max, ss _= peak serum concentration at steady state; C_trough _= mean pre-dose trough concentration on day 24; t_1/2 _= terminal elimination half-life; ANOVA-CV = ANOVA coefficient of variation; Method = method used to calculate 90% CI (ANOVA or Mann-Whitney-Wilcoxon of log-transformed data). *Significant deviation of the ANOVA residuals from normal distribution p < 0.05. The treatments were considered bioequivalent if the ratio and 90% CI fell within the range of 80–125%.

### Pharmacodynamics

**Figure 2:**
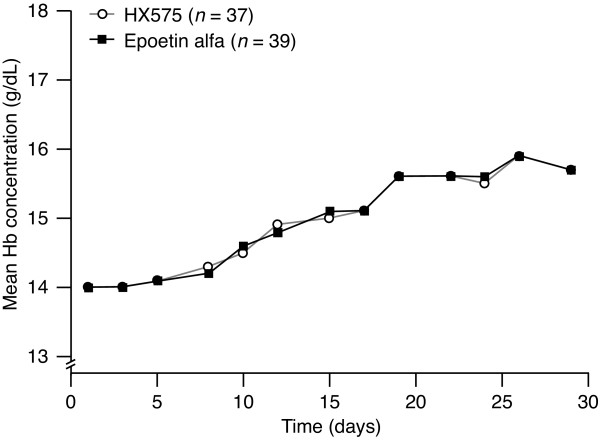
**Mean haemoglobin concentration-versus-time profiles during treatment**.

Both treatment groups had similar baseline Hb concentrations of 14.0 ± 0.6 g/dL (HX575) and 14.0 ± 0.5 g/dL (comparator) and the mean curves were almost congruent (Figure 2). The mean Hb concentrations reached maxima of 15.9 ± 0.8 g/dL (HX575) and 15.9 ± 0.8 g/dL (comparator) after 26 study days. The Hb concentrations had increased from baseline by 1.87 ± 0.64 g/dL (HX575) and 1.97 ± 0.65 g/dL (comparator) on day 26 and by 1.67 ± 0.55/dL (HX575) and 1.76 ± 0.63 g/dL (comparator) on day 29. The Hb concentrations did not further increase after the termination of the treatments.

The AUEC_Hb _was very similar for both treatments (Table 4). The AUEC_Hb _ratio with its 90% CI fell within the stipulated acceptance range of 96.8–103.2%, indicating that the two treatments were bioequivalent in this respect.

**Table 4:** Area under the effect curve (AUEC), AUEC ratio (HX575/comparator), 90% confidence intervals and ANOVA coefficient of variation over days 1–29 of the study for the AUECs of the haematological parameters.

**Haematological parameter**	**HX575**	**Comparator**	**Ratio **(%)	**90% CI **(%)	**ANOVA-CV **(%)
**AUEC_Hb _**(g/dL*h) – GeoM	10049.4	10064.3	99.9	98.5–101.2	3.6

**AUEC_Hb _**(g/dL*h) – GeoCV	3.5%	3.6%	-	-	

**AUEC_RBC _**(10^6^/μL*h) – GeoM	3318	3298	100.6	98.5–102.7	5.4

**AUEC_RBC _**(10^6^/μL*h) – GeoCV	5.5%	5.4%	-	-	

**AUEC_HCT _**(%*h) – GeoM	28796	28912	99.6	98.2–101.0	3.7

**AUEC_HCT _**(%*h) – GeoCV	4.1%	3.3%	-	--	

**AUEC_RET _**(h*10^9^/L) – GeoM	85577	87070	98.3	93.0–103.9	14.5

**AUEC_RET _**(h*10^9^/L) – GeoCV	13.3%	15.6%	-	-	

GeoM = geometric mean; GeoCV = coefficient of variation of GeoM; CI = confidence interval; ANOVA-CV = ANOVA coefficient of variation. Method used to calculate the 90% CI: ANOVA on log-transformed data. The treatments were considered bioequivalent if the ratio and 90% CI of the AUEC_hb _fell within the range of 96.8–103.2%. The other parameters were considered bioequivalent if the respective AUEC ratio and 90% CI fell within the range of 80–125%. Number of subjects analysed: *n *= 37 (HX575) and *n *= 39 (comparator).

**Figure 3:**
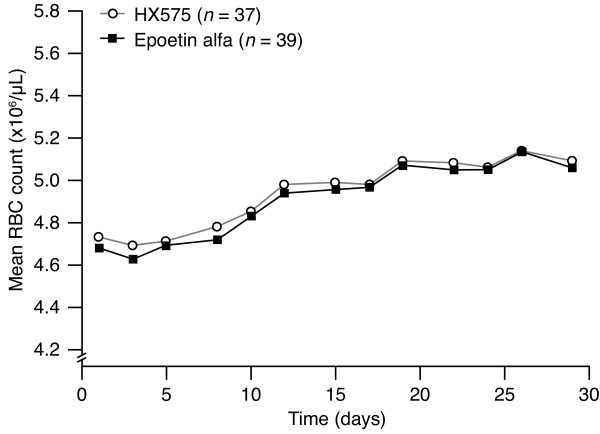
**Mean red blood cell count-versus-time profiles during treatment**.

The mean curves for RBC count were similar for both treatments (Figure 3), starting at similar baseline counts (4.73 ± 0.25*10^6^/μL after HX575 and 4.68 ± 0.26*10^6^/μL after comparator). An increase of RBC counts was observed seven days after the first administration of both treatments. Like the Hb concentrations, the further increase of the mean RBC counts occurred in steps that were synchronous with the application scheme. The RBC counts reached identical maxima of 5.14 ± 0.31*10^6^/μL (HX575) and 5.14 ± 0.30*10^6^/μL (comparator) on study day 26; the increases were 0.41*10^6^/μL (HX575) and 0.46*10^6^/μL (comparator).

The AUEC_RBC _did not show differences between the treatment groups (Table 4). The AUEC_RBC _ratio and 90% CI fell within the standard bioequivalence range of 80–125% (Table 4).

**Figure 4:**
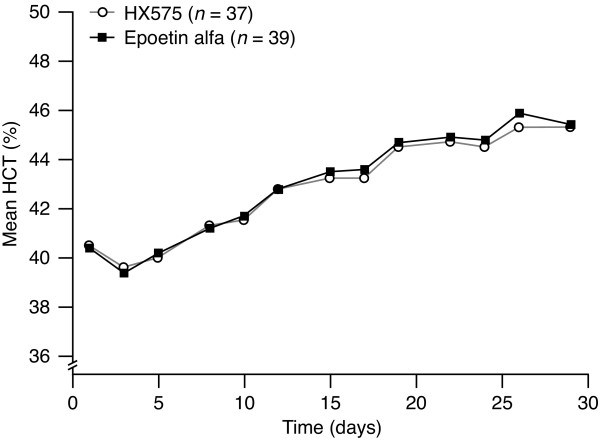
**Mean haematocrit percentage-versus-time profiles during treatment**.

The population mean curves of HCT for the treatments were almost congruent (Figure 4), with similar baseline values (40.5 ± 1.8% after HX575 and 40.4 ± .7% after the comparator). An increase of HCT from baseline was observed eight days after the first application following a transient decrease (probably due to blood loss on study day 1, i.e. baseline). Similar to the Hb concentrations, the further increase of the mean HCT content occurred in steps synchronous with the application scheme. The HCT reached similar maxima of 45.3 ± .2% (HX575) and 45.9 ± 2.0% (comparator) on study day 26, which corresponded to a relative increase of about 11%. The AUEC_HCT _were similar for the two treatments (Table 4). The AUEC_HCT _ratio and 90% CI fell within the standard bioequivalence range of 80–125% (Table 4).

**Figure 5:**
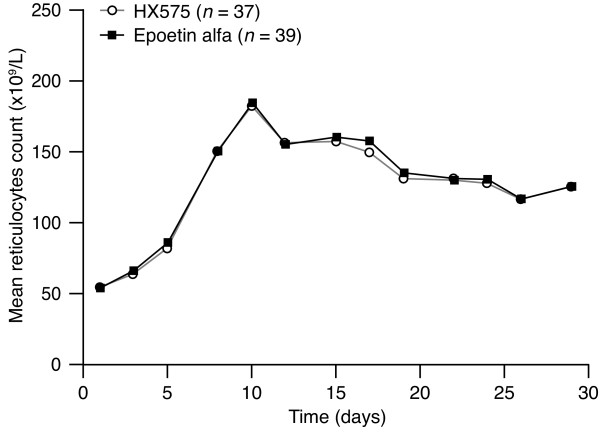
**Mean reticulocyte count-versus-time profiles during treatment**.

The mean curves for Ret count were almost congruent for both treatments (Figure 5) with similar baseline values (54.5 ± 12.6*10^9^/L after HX575 and 54.7 ± 17.7*10^9^/L after the comparator). The increase started with the second application and continued until day 10 to a maximum of 181.9 ± 33.9*10^9^/L (HX575) and 184.7 ± 33.0*10^9^/L (comparator). Thereafter the counts returned step-wise to 125.6 ± 21.8*10^9^/L (HX575) and 126.1 ± 25.3*10^9^/L (comparator) on day 29. The cumulative responses of the reticulocytes to the treatments (AUEC_RET_) were similar (Table 4). The variability of this characteristic was higher than for the RBC counts, but did not differ between the treatments. The AUEC_RET _ratio and 90% CI fell within the standard bioequivalence range of 80–125% (Table 4).

After both treatments the transferrin receptor concentrations increased continuously to more than twice the baseline value (from 1.36 ± 0.44 to 2.86 ± 0.50 mg/L after HX575 and from 1.27 ± 0.40 to 3.04 ± 0.50 mg/L after the comparator). The transferrin concentrations showed only a minor increase compared to baseline (from 2.59 ± 0.47 to 2.75 ± 0.49 g/L after HX575 and from 2.65 ± 0.35 to 2.71 ± 0.36 g/L after the comparator) and returned to baseline levels after the termination of the treatments. A decrease to less than 50% of the baseline value was observed for the ferritin concentration (from 75.9 ± 52.7 to 29.7 ± 15.8 μg/L after HX575 and from 71.9 ± 74.6 to 29.3 ± 22.6 μg/L after the comparator) and for the iron concentration (from 19.6 ± 7.1 to 9.7 ± 3.5 μmol/L after HX575 and from 20.3 ± 8.9 to 8.9 ± 3.8 μmol/L after the comparator). The concentrations returned to the baseline values after termination of the treatments. There was no apparent difference between the treatments.

### Safety

A total of 95 adverse events were reported during the study: 38 events after HX575 compared to 57 events after the comparator. This difference was more pronounced for those events with a suspected relationship to the study medication (13 in the HX575 group versus 31 in the comparator group). However, the clinical relevance of this difference was considered negligible. Of the events with a suspected relationship, arthralgia, headache, tiredness and dizziness were reported most frequently. Anti-epoetin antibodies were not detected in any subject.

The skin tolerability of both preparations was very good; the overall incidence of local reactions was below 2.5%. Skin tolerability of the intravenous HX575 injections (bruising or inflammation at the injection site was observed after five out of 798 injections; 0.6%) was slightly better than that of the comparator (a reaction after 19 out of 827 injections; 2.3%).

## Discussion

The study was designed to estimate the relative bioavailability and to assess bioequivalence with respect to the pharmacodynamic action of the new epoetin formulation, HX575, compared to a registered epoetin alfa formulation with documented efficacy. The primary pharmacokinetic parameter was AUC_τ_. The primary objective of the study was to assess bioequivalence in terms of the AUEC_Hb_. Furthermore, the safety profiles of the two treatments were compared.

The investigational products in this study had similar pharmacokinetic profiles after multiple epoetin doses. The 90% CIs of the ratios for AUC_τ _and C_max, ss _fell within the acceptance range of 80–125%, indicating that HX575 and the comparator epoetin alfa were bioequivalent with respect to the rate and extent of exposure of exogenous epoetin. The treatments were also bioequivalent with respect to the haematological efficacy, as shown by the AUEC_Hb _ratio and 90% CI, which fell within the acceptance range of 96.8–103.2%. The study results thus consistently demonstrate that the haematopoiesis following intravenous dosing was identical after both epoetin formulations. There was no clinically relevant difference in the safety profiles of HX575 and the comparator epoetin alfa.

The C_max _and AUC obtained in the present study agreed well with those obtained in comparable studies with healthy subjects, e.g. Halstenson et al. [21] determined an epoetin alfa baseline-adjusted C_max _of 1796 mIU/mL and an AUC of 12,499 mIU/mL*h in 18 volunteers after a single intravenous dose of 100 mIU/mL.

The magnitudes of the C_max _and AUC after multiple doses were similar to those after single dose. The accumulation ratio for C_max _was 1.2 (HX575) and 1.1 (comparator). The ratio of AUC_τ_/AUC_0–12 _(0.98 after HX575 and 0.89 after the comparator) gives additional evidence that there was no accumulation with respect to the extent of exposure. The true accumulation ratio AUC_0–36, ss_/AUC_0–36, sd _could not be estimated as the time concentration profile was not monitored over a full single dose interval of 36 h. However, the true value of AUC_0–36, ss_/AUC_0–36, sd _must be smaller than that of AUC_0–36, ss_/AUC_0–12_.

The initial pharmacodynamic response after the first doses showed a similar increase of the RBC and the total Ret count for both treatments. There was no indication of any differences between the investigated epoetin formulations with respect to their pharmacodynamic action. The population mean time pharmacodynamic action curves for all investigated pharmacodynamic parameters were congruent.

The increase of the Hb concentrations after repeated doses of epoetin with 1.67 g/dL (HX575) and 1.76 g/dL (comparator) demonstrates that the effect on the haematopoiesis in healthy volunteers was comparable with that observed in patients undergoing haemodialysis [25]. With the recommended epoetin dose and an optimal iron substitution, the increase of the Hb concentration should be 1–2 g/dL per month [26].

## Conclusion

HX575 and the comparator epoetin alfa were bioequivalent in relation to their pharmacokinetic profiles and their pharmacodynamic action at steady state drug concentrations following multiple intravenous administrations with respect to the rate and extent of exposure to ESA. The multiple intravenous doses of the ESA, HX575 and the comparator epoetin alfa were well tolerated with no significant differences between safety profiles of the treatments. HX575 met the bioequivalence criteria with respect to the area under the effect curve of Hb production, which was the primary pharmacodynamic endpoint of this study. These results support the conclusion that HX575, an ESA approved as a biosimilar, and the comparator epoetin alfa, are equally efficacious and may be used interchangeably as intravenous therapy for anaemia in chronic kidney disease.

## Abbreviations

RBC; ESA; AUEC; Hb; Ret; HCT; CI; GeoM; GeoCV

## Competing interests

F. Sörgel has received a fee for speaking, funds for research and consulting fees; U. Thyroff-Friesinger is an employee of Hexal AG and has a small amount of shares in the mother company; A. Vetter is an employee of Hexal AG; B. Vens-Cappell and Martina Kinzig have none to declare. Hexal AG is a subsidiary of Sandoz.

## Authors' contributions

FS, UT-H, AV, BV-C participated in the design and interpretation of the study. BV-C performed the statistical analysis. MK coordinated the study and supervised the analysis of samples. All authors have read and approved the final manuscript.

## Pre-publication history

The pre-publication history for this paper can be accessed here:


